# Supplementary material for: Barriers and facilitators to adherence to secondary stroke prevention medications after stroke: analysis of survivors and caregivers views from an online stroke forum
Source: BMJ Open. 2017 Jul 16;7(7):e016814. doi: 10.1136/bmjopen-2017-016814 (PMC5541606; doi:10.1136/bmjopen-2017-016814)
Supplement: Supplementary file 1 [file bmjopen-2017-016814supp001.pdf]

Supplementary File 1. Analysis strategy to identify study posts

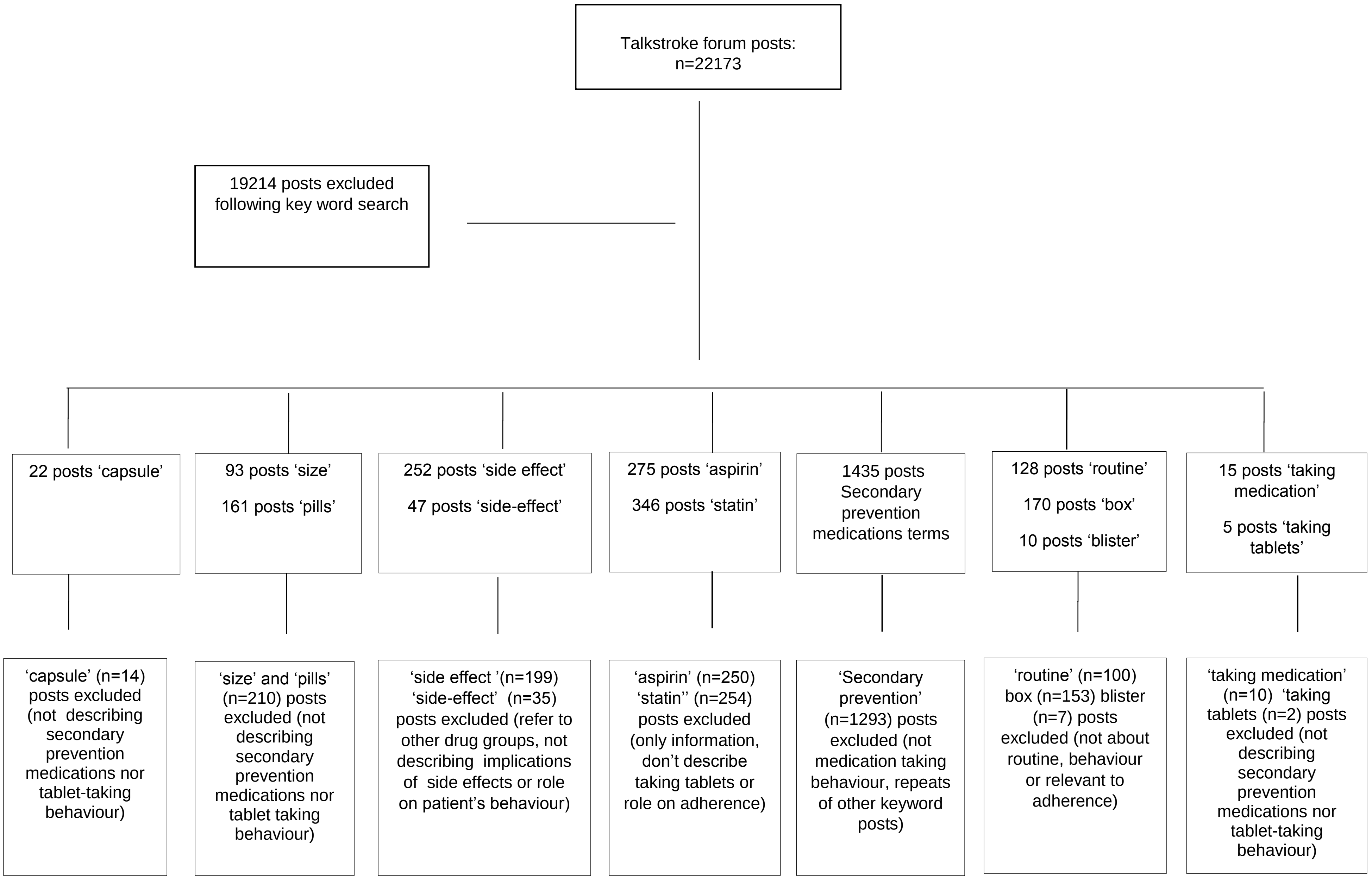

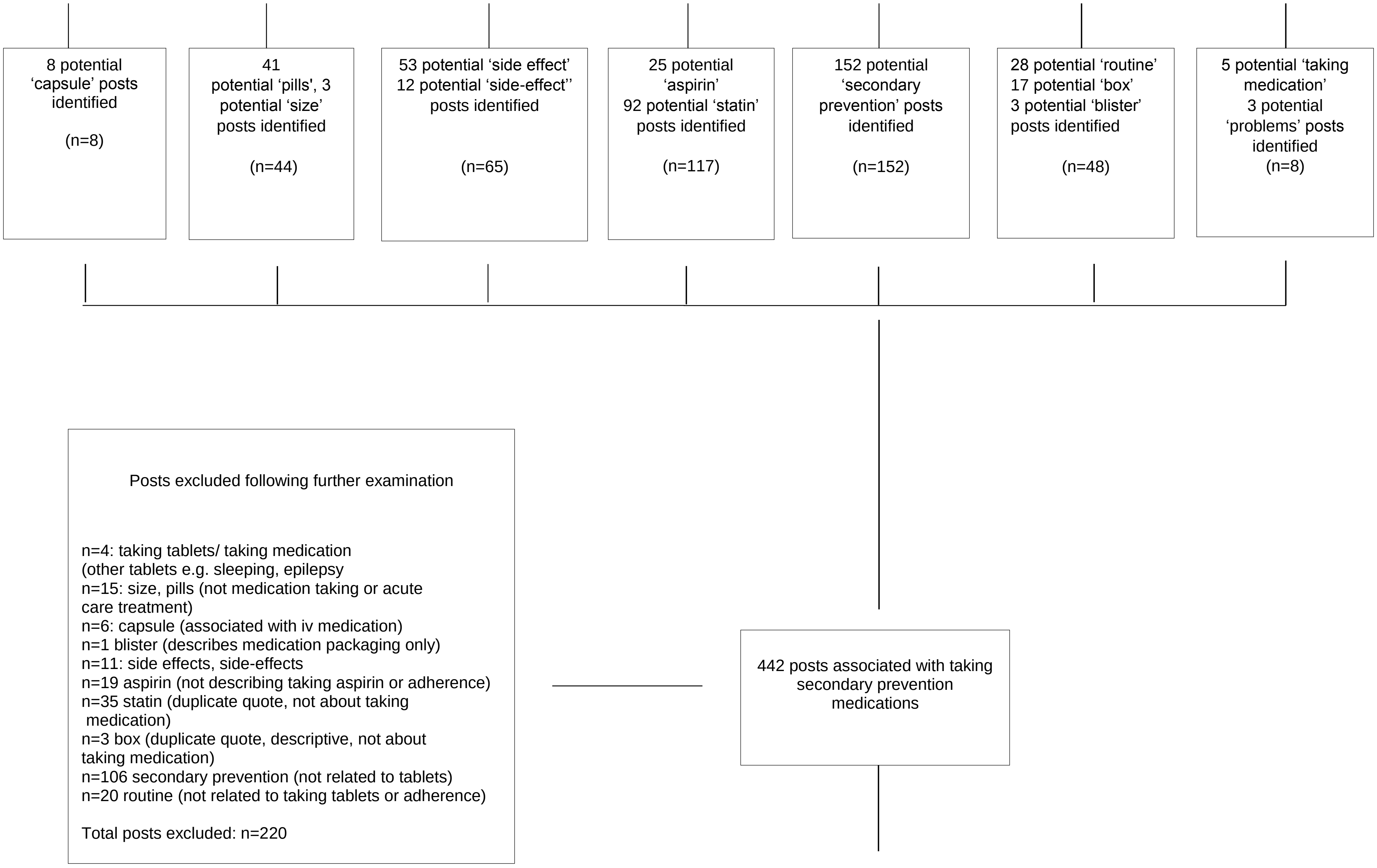
8 potential ‘capsule’ posts identified
(n=8)
41 potential ‘pills', 3 potential ‘size’ posts identified
(n=44)
53 potential ‘side effect’ 12 potential ‘side-effect’’ posts identified
(n=65)
25 potential ‘aspirin’ 92 potential ‘statin’ posts identified
(n=117)
152 potential ‘secondary prevention’ posts identified
(n=152)
28 potential ‘routine’ 17 potential ‘box’ 3 potential ‘blister’ posts identified
(n=48)
5 potential ‘taking medication’ 3 potential ‘problems’ posts identified
(n=8)
Posts excluded following further examination
n=4: taking tablets/ taking medication (other tablets e.g. sleeping, epilepsy
n=15: size, pills (not medication taking or acute care treatment)
n=6: capsule (associated with iv medication)
n=1 blister (describes medication packaging only)
n=11: side effects, side-effects
n=19 aspirin (not describing taking aspirin or adherence)
n=35 statin (duplicate quote, not about taking medication)
n=3 box (duplicate quote, descriptive, not about taking medication)
n=106 secondary prevention (not related to tablets)
n=20 routine (not related to taking tablets or adherence)
Total posts excluded: n=220
442 posts associated with taking secondary prevention medications

Posts analysed: thematic analysis on themes classified as barriers or facilitators of medication adherence.

n= 4: Taking medication/ taking tablets
n= 29: Size, pills
n= 2: Capsule
n= 2: Blister
n= 54: Side effects, side-effects
n= 14: Box
n=109: Secondary prevention medication terms
n= 8: routine

Total posts included: n= 222

Thematic analysis: Development of themes associated with barriers and facilitators of medication adherence
